# Split Ring Antennas and Their Application for Antenna Miniaturization

**DOI:** 10.3390/s23020846

**Published:** 2023-01-11

**Authors:** Yanxia Liu, Lotfollah Shafai, Dustin Isleifson, Cyrus Shafai

**Affiliations:** 1 Department of Electrical & Computer Engineering, University of Manitoba, Winnipeg, MB R3T 5V6, Canada; 2 Centre for Earth Observation Science, University of Manitoba, Winnipeg, MB R3T 2N2, Canada

**Keywords:** antenna miniaturization, dielectric resonator antenna, split ring resonator, modified split ring resonator

## Abstract

This paper investigates the miniaturization capability of split ring array antennas embedded in a low-permittivity dielectric substrate, in comparison with the same-sized high-permittivity dielectric resonator antennas (DRAs). In order to understand the miniaturization performance, a size-fixed dielectric substrate with different split ring arrays is studied. The simulation results show that the miniaturization capability increases with decreased unit cell resonant frequency and/or increased unit cell induced permeability. Miniaturizations as high as 25.54 times that of a high-permittivity DRA are obtained with split rings, etched on a dielectric substrate having a low permittivity of 2.2. Furthermore, this excessive miniaturization does not come at the expense of excessive deterioration of the antenna impedance bandwidth, gain, and radiation efficiency. Consequently, the miniaturized split ring arrays still provide high gains over wider bandwidths. This inference is further verified by comparing the miniaturization and other antenna performance parameters with three other modified split ring configurations. To experimentally verify this work, a split ring antenna was fabricated and tested, and good agreement between the simulated and measured results was observed. The results of this study indicate that adding resonant metallic inclusions into low- permittivity DRAs significantly increases their miniaturization capability, without overly deteriorating the performance.

## 1. Introduction

The trend for smaller and lighter electronic devices continues to drive the need for miniaturized antennas and electronic components. However, antenna size reduction usually comes with a sacrifice in antenna performance, such as reduced radiation efficiency, narrowed bandwidth, and difficulty in impedance matching [1,2]. Thus, the essential problem with antenna miniaturization is to develop an appropriate technique that allows the miniaturized antenna to work as well as, or to be at least comparable to, a normal- sized antenna. Such requirements are especially important in programmable surfaces and metasurfaces that demand miniaturized antennas for communication and sensing applications [3,4,5]. From the material point of view, an antenna can be miniaturized by using natural or artificial high-permittivity and/or high-permeability materials. In studies [6,7], dielectric resonator antennas (DRAs) have been successfully miniaturized by using a natural and an artificial high-permittivity material, respectively [8,9,10,11,12,13]. It has been reported that an antenna with high-permittivity materials provides less bandwidth compared to the same electrical-sized antenna with high-permeability materials [14,15]. However, naturally occurring high-permeability materials are too lossy for low-loss microwave applications [16]. Therefore, artificially engineered high-permeability materials have been chosen for applications that operate in the microwave frequency band [16,17].

In a permanent magnet, aside from the nuclear and electron spins, orbiting electrons create currents that circulate around the nuclei, and these currents, in turn, create magnetic dipole moments along the axes of the current loops. This allows the magnet to have high permeability [18]. Similarly, high permeability can be induced in an otherwise non-magnetic material if circulating currents can be created [19]. In study [16], permeability was effectively induced in a dielectric by embedding loop-like metallic inclusions in the material. However, the electromagnetic interaction between the loops and the magnetic field only occurs when they are orthogonal [16,20]. Since the key to successful permeability induction is circulating currents, the metallic inclusions should be loop-like so that circulating currents can flow in them. A variety of such loops have been reported in the literature, such as split ring resonator (SRR), complementary split ring resonator (CSRR), multiple split ring resonator (MSRR), spiral resonator (SR), and extended split ring resonator [19,21,22].

In the present study, 3-dimensional arrays of SRRs are used to generate high-permeability materials. For ease of fabrication, low-loss thin layers of microwave substrates are used to etch the SRR arrays on their surface. Stacking multiple layers are then used to further amplify the permeability-inducing effects. In such an antenna configuration, the excitation must generate a magnetic field perpendicular to the SRR array surface to generate high permeability. Such an antenna concept and its configurations are presented in Section 2.1. The miniaturization capability of the design and its detailed performance are also presented in Section 2.1. Section 2.2 compares the performance of the 3-dimentional arrays of different types of split rings. In order to verify the simulation results, an antenna prototype was fabricated and tested, and the results are discussed in Section 3. Conclusions are presented in Section 4, indicating that significantly high antenna miniaturizations can be obtained by using low-permittivity DRAs, without sacrificing their performance.

## 2. Antenna Configuration

### 2.1. 3-Dimensional Split Ring Array

**Figure 1:**
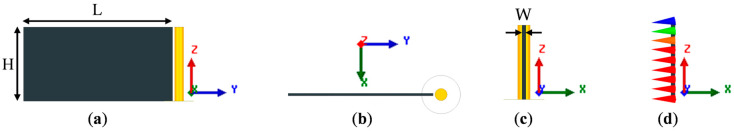
Geometry of the low-loss host substrate of width (*W*), length (*L*), and height (*H*): (**a**) Front view (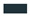 Host substrate, 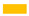 Probe); (**b**) Top view; (**c**) Left side view; (**d**) Excitation magnetic field in the substrate. (Ground plane not shown in all four sub-figures, and probe not shown in (**d**)).

The geometry of the host substrate is shown in Figure 1. It is a thin low-loss dielectric material of thickness *w* = 0.254 mm, length *L* = 15 mm, and height *H* = 7.5 mm, made of two identical dielectric layers of thickness 0.127 mm stacked along the *X*-axis. The excitation feed is a vertical probe of 0.625 mm in radius (i.e., $r_{\mathrm{probe}}$ = 0.625 mm) and 7.5 mm in height ($h_{\mathrm{probe}}$ = 7.5 mm). It is fed by an SMA connector, having the inner pin radius of 0.625 mm and outer conductor radius of 2 mm (i.e., $r_{\mathrm{port}}$ = 2 mm). The probe is placed vertically, parallel to the edge of the substrate. For this geometry, the magnetic field generated by the probe is perpendicular to the surface of the substrate. The specifications of the host substrate and excitation probe are shown in Table 1.

**Figure 2:**
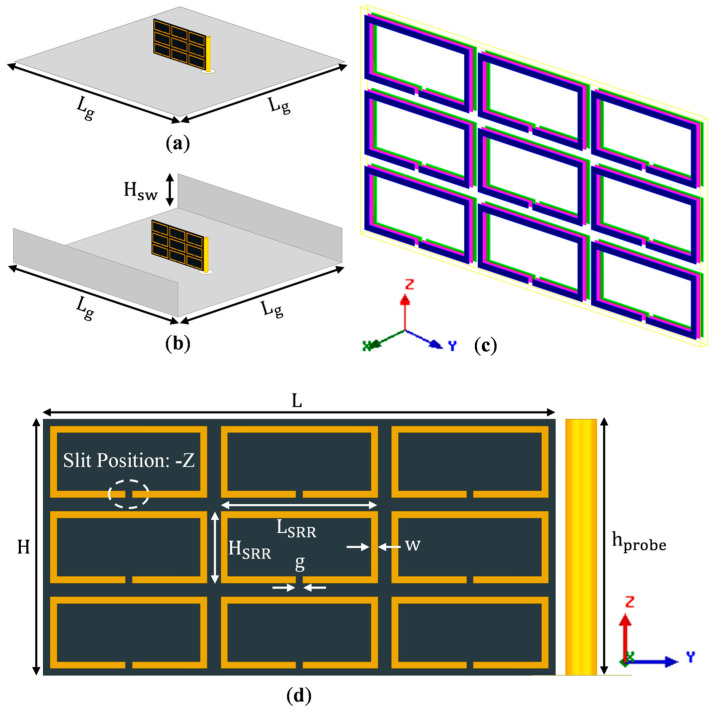
(**a**) Oblique view of an SRR array embedded in the host substrate with 3 elements in all 3 directions of X, Y, and Z, denoted as$\;3\vec{x}\times 3\vec{y}\times 3\vec{z}$, placed on a conventional planar ground plane (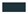 Host substrate; 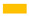 SRRs and probe; 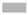 Ground plane). (**b**) Oblique view of the same antenna on a U-shaped ground plane. (**c**) Zoomed-in view of the ring arrangements on the substrate (different colors are used to demonstrate different layers of SRRs). The three identical layers of SRRs are placed on the front (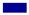 blue), center (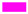 pink), and back (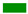 green) surfaces. (**d**) Zoomed-in front view of the SRR array with dimensions as marked.

The idea of etching the SRR array antenna on the substrate (dimensions given in Table 1 and Figure 1) is to effectively miniaturize the antenna by artificially inducing permeability in the host dielectric. Since the magnetic field is along the *X*-axis (see Figure 1d), the rings should be placed in the YZ-plane to maximize the electromagnetic interaction between the split rings and the applied magnetic field [16]. The antenna geometry is shown in Figure 2.

The miniaturization capability (MC) of the split ring arrays in this work is defined as the ratio of the refractive index of the host substrate with split ring arrays (i.e., artificial material), i.e., $n_{artificial}$, to that of the same host substrate without split ring arrays, i.e., $n_{host}$. That is:

(1) $$MC=\frac{n_{artificial}}{n_{host}}$$

Since the host substrate is a dielectric with known material properties (i.e., $\varepsilon_{r\_\mathrm{host}}$ = 2.2, and $\mu_{r\_\mathrm{host}}$ = 1), its refractive index can be simply expressed as:

(2) $$n_{host}=\sqrt{\varepsilon_{r\_host}{*\mu }_{r\_\mathrm{host}}}=\sqrt{\varepsilon_{r\_host}}=\sqrt{2.2}$$

In order to find the refractive index of the host substrate with split rings, a reference dielectric resonator antenna (DRA), with the exact same physical dimensions and resonant frequency as those of the split ring array antennas, needs to be introduced. We call this reference antenna “equivalent high-ε DRA” or “equivalent DRA”. The refractive index of the split ring array antenna, $n_{artificial}$, is equal to that of the equivalent DRA, $n_{equivalent}$. Since the equivalent DRA is purely made of a dielectric material with relative permittivity $\varepsilon_{r\_equivalent}$, its refractive index is given by:

(3) $$n_{equivalent}=\sqrt{\varepsilon_{r\_equivalent}}$$

With known physical dimensions and resonant frequency, the equivalent relative permittivity ($\varepsilon_{r\_equivalent}$) of such an equivalent DRA can be both numerically simulated in ANSYS © HFSS and theoretically calculated using the equations in [23]. Therefore, Equation (1) can be rewritten as:

(4) $$MC=\frac{n_{loaded}}{n_{host}}=\frac{n_{equivalent}}{n_{host}}=\frac{\sqrt{\varepsilon_{r\_equivalent}}}{\sqrt{\varepsilon_{r\_host}}}$$

which is used to assess the miniaturization capability of the split ring antennas in this work.

Figure 2 demonstrates a 27-element split ring array antenna, in which three identical layers of 9-element split ring arrays are placed at the front, center, and back, etched on two identical host dielectric substrates, one with metallization on one side and the other with metallization on both sides. The 3-layer split ring arrays and 2-layer host substrates are stacked along the *X*-axis, forming a 5-layer sandwich structure. The impedance matching of this antenna can be controlled by adjusting the height of the probe ($h_{probe}$), the spacing between the dielectric and the probe ($g_{probe}$), or both. As the antenna is capacitive, increasing the probe length adds inductance to the antenna and makes the antenna resonate at a lower frequency. Meanwhile, with the addition of inductance to a capacitive antenna, a larger portion of the Smith Chart loop falls into the −10 dB matching circle and results in a wider impedance bandwidth [24]. However, since the probe is a monopole radiating in the horizontal direction, increasing its length reduces the antenna directivity in broadside direction, and increases its cross-polarization [24]. Therefore, the probe height is chosen to be the same as the substrate height, i.e., $h_{probe}$ = *H* = 7.5 mm, and its spacing from the substrate is used to adjust S11.

For further control of the directivity and cross-polarization, a U-shaped ground plane (Figure 2b), as opposed to a conventional planar ground plane (Figure 2a), is used to counteract the probe effects [25,26,27]. Directivity and cross-polarization also depend on both ground plane length,$\;L_{g}$, and sidewall height, $H_{sw}$. Their optimal sizes for the highest directivity and lowest cross-polarization are found to be $L_{g}=1.2\lambda$ and$\;H_{sw}=0.4\lambda$ [26,27].

For a size-fixed substrate, varying the ring length ($L_{SRR}$) or ring height ($H_{SRR}$) leads to different ring arrangements, which result in different induced material properties and thus different miniaturization performance. Therefore, the effect of ring length and height are studied for different split ring arrangements. By varying the dimensions of the split rings, numerous ring arrangements can be obtained, six of which, as listed in Table 2, were investigated. To simplify the analysis, the ring arrangement along the *X*-axis remains the same and only varies in the YZ-plane. In this study, a 3-layer configuration (i.e.,$\;3\;\vec{x}$) is used, and these three identical layers of split rings are placed at the front, center, and back. For the sake of convenience, the six ring arrangements are referred to as: $3\;\vec{x}\times 3\;\vec{y}\times 3\;\vec{z}$, $3\;\vec{x}\times 3\;\vec{y}\times 2\;\vec{z}$, 3$\;\vec{x}\times 3\;\vec{y}\times 1\;\vec{z}$, $3\;\vec{x}\times 2\;\vec{y}\times 2\;\vec{z}$, $3\;\vec{x}\times 2\;\vec{y}\times 1\;\vec{z}$, and$\;3\;\vec{x}\times 1\;\vec{y}\times 1\;\vec{z}$, in which the numbers represent the number of split rings in three specific directions, namely, *X*-axis ($\vec{x}$), *Y*-axis ($\vec{y}$), and *Z*-axis ($\vec{z}$).

Split rings are placed at a spacing, s, away from each other along the Y- and Z-axes. In other words, each split ring layer in the YZ-plane is made of repetitive split ring unit cells. The dimensions of the split ring unit cells for each arrangement are given in Table 2, which are calculated by using the following equations:

*L* = 15 mm, *H* = 7.5 mm → *L* = 2*H* (5)

(6) $$N_{L}=\frac{L}{L_{unit\;cell}},\;N_{H}=\frac{H}{H_{unit\;cell}}$$

(7) $$\frac{N_{L}}{N_{H}}=\frac{L}{L_{unit\;cell}}\frac{H_{unit\;cell}}{H}=2\frac{H_{unit\;cell}}{L_{unit\;cell}}$$

(8) $$N_{L}:N_{H}=3:3,\;2:2,\;and\;1:1\to L_{unit\;cell}=2H_{unit\;cell}$$

(9) $$N_{L}:N_{H}=3:2\to L_{unit\;cell}=\frac{4}{3}H_{unit\;cell}$$

(10) $$N_{L}:N_{H}=3:1\to L_{unit\;cell}=\frac{2}{3}H_{unit\;cell}$$

(11) $$N_{L}:N_{H}=2:1\to L_{unit\;cell}=1H_{unit\;cell}$$

(12) s=0.4 mm

(13) $$L_{SRR}=L_{unit\;cell}-s$$

(14) $$H_{SRR}=H_{unit\;cell}-s$$

where $N_{L}$ and $N_{H}$ are ring numbers along the Y- and Z-axes, respectively, s is the spacing between adjacent split rings in the YZ-plane. $L_{SRR}$ and $L_{unit\;cell}$ are the lengths of split rings and split ring unit cells along the *Y*-axis, respectively, and $H_{SRR}$ and $H_{unit\;cell}$ are the heights of split ring and split ring unit cells along the *Z*-axis, respectively.

For the ring arrangement study, the ring width, gap width, and spacing between adjacent rings remain the same, namely, w=0.2 mm, g=0.2 mm, and s=0.4 mm. The miniaturization capability of a split ring array is controlled by two parameters, namely, the peak relative permeability and the resonant frequency of the corresponding split ring unit cell. The resonant frequency roughly sets the operating frequency range of the engineered material [19], and the peak relative permeability simply determines how strong the magnetic strength of the material is. In order to improve the miniaturization capability (MC), one should either reduce the resonant frequency of the unit cell or increase the peak relative permeability of the unit cell, or preferably both.

The resonant frequency of the unit cell is inversely proportional to the circumference of the split ring, $C_{SRR}$. The peak relative permeability is proportional to the product of the ring number *N*, current *I*, and effective ring area *A*, i.e., *NIA.* Since the width of the rings for different arrangements remains the same, i.e., w=0.2 mm, the current is assumed to be approximately the same in all six cases, which means the only term that makes a difference in the peak relative permeability of the engineered materials with different ring arrangements is *NA*. As can be seen from Table 2, both *NA* and $C_{SRR}$ vary with the ring arrangement, which means both the resonant frequency and the peak relative permeability of the unit cell vary with the ring arrangement. More specifically, the resonant frequency ($f_{uc}$) and the relative permeability ($\mu_{r}$) of the unit cell for different ring arrangements vary with the following orders, respectively: $f_{\mathrm{uc}\_3\;\vec{x}\times 3\;\vec{y}\times 3\;\vec{z}}$ > $f_{\mathrm{uc}\_3\;\vec{x}\times 3\;\vec{y}\times 2\;\vec{z}}$ > $f_{\mathrm{uc}\_3\;\vec{x}\times 2\;\vec{y}\times 2\;\vec{z}}$ > $f_{\mathrm{uc}\_3\;\vec{x}\times 3\;\vec{y}\times 1\;\vec{z}}$ > $f_{\mathrm{uc}\_3\;\vec{x}\times 2\;\vec{y}\times 1\;\vec{z}}$ > $f_{\mathrm{uc}\_3\;\vec{x}\times 1\;\vec{y}\times 1\;\vec{z}}$; and $\mu_{r\_3\;\vec{x}\times 3\;\vec{y}\times 3\;\vec{z}}$ < $\mu_{r\_3\;\vec{x}\times 3\;\vec{y}\times 2\;\vec{z}}$ < $\mu_{r\_3\;\vec{x}\times 2\;\vec{y}\times 2\;\vec{z}}$ < $\mu_{r\_3\;\vec{x}\times 3\;\vec{y}\times 1\;\vec{z}}$ < $\mu_{r\_3\;\vec{x}\times 2\;\vec{y}\times 1\;\vec{z}}$ < $\mu_{r\_3\;\vec{x}\times 1\;\vec{y}\times 1\;\vec{z}}$. Therefore, the miniaturization capabilities (MCs) of the six ring arrangements should be in the following order: ${\mathrm{MC}}_{3\;\vec{x}\times 3\;\vec{y}\times 3\;\vec{z}}$ < ${\mathrm{MC}}_{3\;\vec{x}\times 3\;\vec{y}\times 2\;\vec{z}}$ < ${\mathrm{MC}}_{3\;\vec{x}\times 2\;\vec{y}\times 2\;\vec{z}}$ < ${\mathrm{MC}}_{3\;\vec{x}\times 3\;\vec{y}\times 1\;\vec{z}}$ < ${\mathrm{MC}}_{3\;\vec{x}\times 2\;\vec{y}\times 1\;\vec{z}}$ < ${\mathrm{MC}}_{3\;\vec{x}\times 1\;\vec{y}\times 1\;\vec{z}}$, which will be verified in this section through simulations.

**Figure 3:**
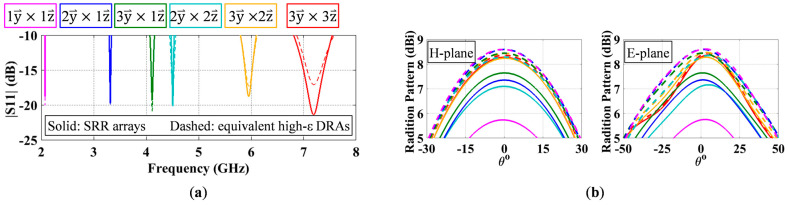
(**a**) S11 results of SRR arrays (solid) with different SRR arrangements and their corresponding equivalent high-ε DRAs (dashed). (**b**) E- and H-plane radiation patterns of SRR arrays with different SRR arrangements (solid: realized gain; dashed: directivity). Different colors represent different SRR arrangements: 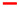 $3\;\vec{y}\times 3\;\vec{z}$, 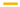 $3\;\vec{y}\times 2\;\vec{z}$, 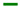 $3\;\vec{y}\times 1\;\vec{z}$, 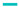 $2\;\vec{y}\times 2\;\vec{z}$, 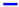 $2\;\vec{y}\times 1\;\vec{z}$, 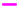 $1\;\vec{y}\times 1\;\vec{z}$.

Figure 3a shows the S11 results of the SRR array antennas with different ring arrangements, represented by different colors. As expected, the resonant frequency varies with the ring arrangement. To establish a comparison basis for the miniaturization capability of different ring arrangements, their performance is compared with that of identical- sized equivalent high-ε DRAs. In this manner, making the resonant frequency of an equivalent DRA match that of a split ring array, the ratio of their refractive indices will be a good indication of its miniaturization capability. To do this, one first needs to find relative permittivity values of the six equivalent high-ε DRAs whose resonant frequencies (dashed curves in Figure 3a) match those of the six split ring arrays antennas (solid curves in Figure 3a). Since the dimensions and the resonant frequencies of the equivalent DRAs are known, the equivalent relative permittivities, $\varepsilon_{r\_equivalent}$, can be both numerically simulated in ANSYS © HFSS and theoretically calculated using the equations in [23]. Solid curves in Figure 3a are the S11s of the six SRR arrays with different ring arrangements, and each color represents a specific arrangement. The S11 of each equivalent high-ε DRA is shown in Figure 3a as a dashed curve of the same color as its SRR array counterpart. Both simulated (i.e., Sim.) and calculated (i.e., Cal.) equivalent relative permittivities are given in Table 3. For miniaturization capability evaluation, the simulated values were used. As illustrated in Table 3, the simulated equivalent relative permittivities of ring arrangement are$\;3\;\vec{x}\times 3\;\vec{y}\times 3\;\vec{z}$, $3\;\vec{x}\times 3\;\vec{y}\times 2\;\vec{z}$, $3\;\vec{x}\times 2\;\vec{y}\times 2\;\vec{z}$, $3\;\vec{x}\times 3\;\vec{y}\times 1\;\vec{z}$, $3\;\vec{x}\times 2\;\vec{y}\times 1\;\vec{z}$, and $3\;\vec{x}\times 1\;\vec{y}\times 1\;\vec{z}$ are 90.0, 143.7, 273.8, 335.5, 536.5, and 1435.0, respectively, and the corresponding miniaturization capabilities are 6.40, 8.08, 11.16, 12.35, 15.62, and 25.54, respectively, following the same miniaturization capability order as predicted. This verifies that the miniaturization capability of an artificial magneto-dielectric material indeed is dependent on two factors, namely, resonant frequency and relative peak permeability of the unit cell. More specifically, a lower unit cell resonant frequency and/or a higher relative peak permeability result in a greater miniaturization capability. For instance, among the six arrangements, the unit cell of arrangement $3\;\vec{x}\times 3\;\vec{y}\times 3\;\vec{z}$ has the highest resonant frequency due to its shortest split ring circumference and lowest relative peak permeability due to its lowest *NA* value, and thus the SRR array of $3\;\vec{x}\times 3\;\vec{y}\times 3\;\vec{z}$ is the least miniaturized. On the contrary, the unit cell of arrangement $3\;\vec{x}\times 1\;\vec{y}\times 1\;\vec{z}$ has the lowest resonant frequency due to its longest circumference and highest relative peak permeability due to its highest *NA* value, and thus the SRR array of $3\;\vec{x}\times 1\;\vec{y}\times 1\;\vec{z}$ is the most miniaturized.

Figure 3b shows the directivities (dashed) and realized gains (solid) of the SRR array antennas with different ring arrangements. The detailed antenna performance of these SRR arrays is given in Table 3. As antennas become more miniaturized, reduced efficiency (η), gain, and/or bandwidth (BW) are expected due to the fundamental limitations of small antennas [1,2]. It has been demonstrated in [24,26] that one can increase the ring trace width w and/or gap width g, to improve the radiation efficiency of a miniaturized SRR array, providing there is still room for improvement. It is interesting to notice that the performance degradation in some arrangements is more significant than others. In this case, the arrangement $2\;\vec{y}\times 2\;\vec{z}$ exhibits the highest performance degradation. More specially, compared to the case of $3\;\vec{x}\times 2\;\vec{y}\times 2\;\vec{z}$, the case of$\;3\;\vec{x}\times 3\;\vec{y}\times 1\;\vec{z}$, despite being more miniaturized, has higher radiation efficiency, higher gain, lower cross-polarization, and wider impedance bandwidth. This is due to the fact that $3\;\vec{x}\times 2\;\vec{y}\times 2\;\vec{z}$ has more conductor length (238.8 mm), and thus higher loss than$\;3\;\vec{x}\times 3\;\vec{y}\times 1\;\vec{z}$ (201.6 mm).

### 2.2. Arrays with Modified Split Ring Geometries

**Figure 4:**
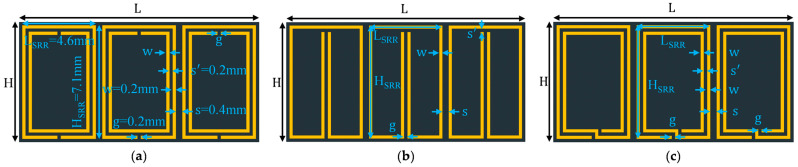
Host substrate with $3\;\vec{x}\times 3\;\vec{y}\times 1\;\vec{z}$ arrays of modified SRRs: (**a**) complimentary SRRs (CSRRs); (**b**) SRRs with extended arms (ESRRs); (**c**) spiral SRRs (SSRRs) (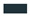 Host substrate (*L* = 15 mm, *H* = 7.5 mm); 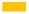 Modified SRRs. Ground plane and probe are not shown.

**Figure 5:**
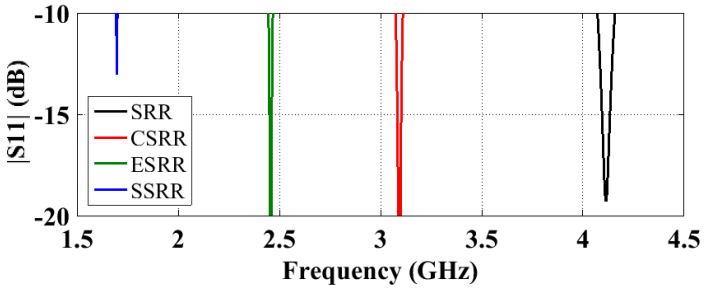
S11 results of $3\;\vec{x}\times 3\;\vec{y}\times 1\vec{z}$ antennas with different SRR geometries: black, conventional SRR; red, CSRR; green, ESRR, and blue, SSRR.

In this section, we investigate the performance of the SRR arrays with three new split ring geometries to examine their miniaturization performance. The arrangement of$\;3\vec{x}\times 3\vec{y}\times 1\vec{z}$, as shown in Figure 4, was selected for this study. Since all three modified SRRs, namely, complimentary SRR (CSRR), SRR with extended arms (ESRR), and spiral SRR (SSRR), are inductively and/or capacitively loaded versions of a conventional SRR [19], a unit cell of any of them should have a lower resonant frequency than an SRR unit cell of the same dimensions. Simulation shows that the resonant frequencies of the three modified SRR unit cells are indeed lower than that of the conventional SRR unit cell, and the four unit cell resonant frequencies are in the sequential order of $f_{\mathrm{uc}\_\mathrm{SRR}}$ > $f_{\mathrm{uc}\_\mathrm{CSRR}}$ > $f_{\mathrm{uc}\_\mathrm{ESRR}}$ > $f_{\mathrm{uc}\_\mathrm{SSRR}}$. Therefore, based on the analysis, the resonant frequencies of the arrays with the four different SRRs should follow the same order, which is verified by the S11 results in Figure 5. Table 4 shows the detailed performance of their antennas. As expected, bandwidth (BW), radiation efficiency (η), and gain reduction are observed as the antennas become more miniaturized. Similar to the conventional SRR arrays, the radiation efficiency of modified SRR arrays can be improved by increasing the trace width and gap width of the inclusions as demonstrated in Table 4, except for the one with CSRRs.

## 3. Fabrication and Measurement

**Figure 6:**
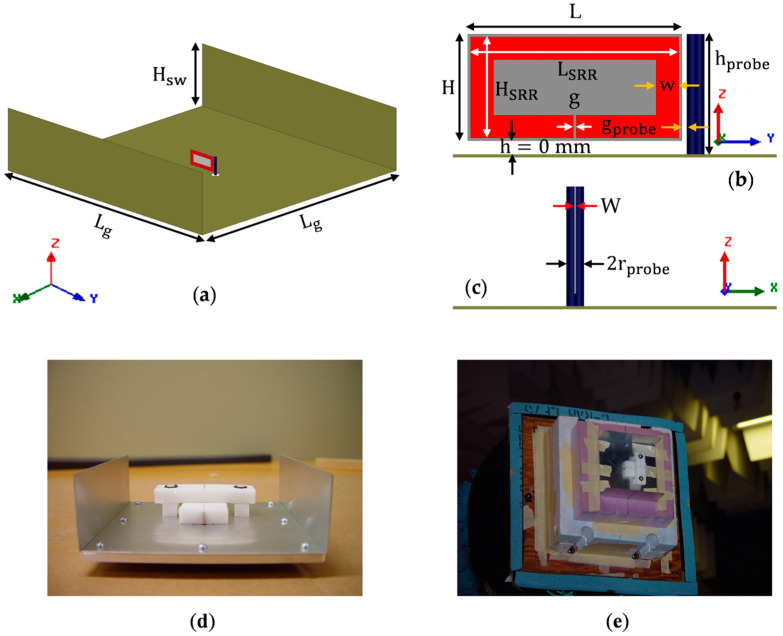
Fabricated $1\;\vec{x}\times 1\;\vec{y}\times 1\;\vec{z}$ split ring antenna. The split ring is placed on the front surface of the dielectric. Each color represents one part of the antenna: 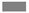 Host substrate; 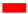 SRR; 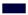 Probe; 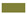 Ground plane. (**a**) Oblique, (**b**) front and (**c**) side views of the simulated antenna. (**d**) Oblique view of the fabricated antenna and (**e**) the fabricated antenna under test, mounted on the tower.

In order to experimentally verify the above studies, a split ring antenna, as shown in Figure 6, was fabricated and tested. The dimensions of the antenna are given in Table 5. The antenna is based on the same low-permittivity substrate that is used throughout this work, i.e., Rogers RT/duroid 5880 ($\varepsilon_{r\_\mathrm{host}}=2.2,{\;\tan\delta }_{\mathrm{host}}=0.0009$, and thickness of 0.127 mm). The ring arrangement of $1\;\vec{x}\times 1\;\vec{y}\times 1\;\vec{z}$ was chosen, as from the previous discussions. The ring arrangement $1\;\vec{y}\times 1\;\vec{z}$ in the YZ-plane can provide the highest miniaturization capability among all the arrangements that have been studied. A milling machine was used to remove the excess copper from the substrate to make the split ring, which had a metallization width of 1.6 mm. Removing so much copper from the surface of such a thin substrate made it too flexible, and it was impossible to do the same on both sides. It should also be mentioned that using a milling machine for the process of removing copper from the substrate surface also removes a thin layer of the dielectric, which makes it even more flexible. Thus, in fabrication, a single-layer substrate with one-sided metallization was used.

Since the substrate was too flexible to stay vertical, it was sandwiched between two foam blocks using double-sided scotch tape and nylon screws. Additional foam blocks and nylon screws were then used to hold it down firmly on the ground plane. To restrain the movement of the probe, we used a thick ground plane in the fabricated unit. The completed fabrication model is shown in Figure 6a–d. Furthermore, in measuring the radiation patterns, the finite size of the ground plane can cause coupling with the mounting tower. To eliminate such coupling effects, the fabricated model was lifted above the tower head, using about one wavelength-thick foams and taped, to eliminate any scattering sources. The mounted unit on the tower is shown in Figure 6e.

**Figure 7:**
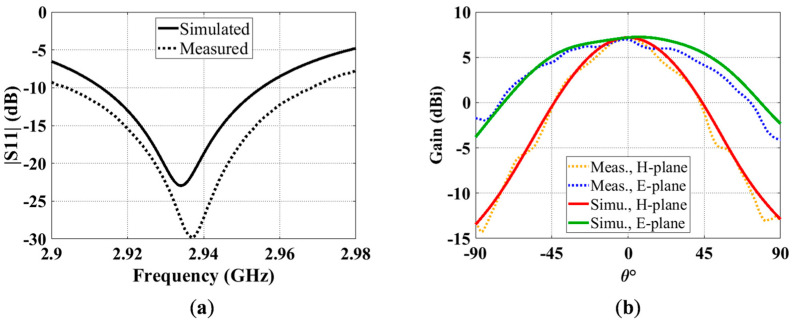
Simulated (solid) and measured (dashed) results of the fabricated split ring antenna: (**a**) S11; (**b**) E- and H-plane radiation patterns at 2.937 GHz.

The simulated and measured S11 results of the fabricated antenna are compared in Figure 7a. As can be seen, the measured (2.937 GHz) and simulated (2.934 GHz) resonant frequencies agree well. In measurement, a wider bandwidth of 2.21% was achieved, while the simulated bandwidth is 1.44%. There are multiple important factors that could have contributed to the discrepancy between the two bandwidths. For example, in the fabricated model, the shaving effect on the dielectric due to the milling machine reduced the substrate thickness to around 0.109 mm, while the original substrate thickness was 0.127 mm. In addition, the extra materials used for stabilizing the antenna, i.e., double- sided tape, foam blocks, and nylon screws, added additional dielectric with higher losses to the fabricated antenna. Additionally, the thick ground plane in the fabricated unit provided a short transition for impedance matching. Since the fabricated antenna was glued between two foam blocks and then placed on the ground plane, ensuring h = 0 mm along the entire length *L* of the antenna was difficult and uncontrollable, as the foam blocks prevented a visual view of the antenna. Unfortunately, due to having too many unknowns and too many combinations, it was challenging to find the exact combination of parameters that caused the measured bandwidth to become wider. Figure 7b demonstrates the comparison of the simulated and measured E- and H-plane radiation patterns at 2.937 GHz. The measured and simulated gains also agree well, which are about 7.15 and 7.19 dBi, respectively.

## 4. Conclusions

This work investigated the miniaturization capability of split ring array antennas that are etched on a low-permittivity dielectric substrate. Compared to conventional pure dielectric-based DRAs, they are sturdier and more compact. Based on the ring arrangement study, an analysis on the relationship between the miniaturization capability and ring parameters was made. More specifically, the lower the unit cell resonant frequency and/or the higher the induced peak permeability, the higher the miniaturization capability. This inference was further verified by comparing one of the split ring array antennas with three modified split ring array antennas. Similar results were obtained. Finally, the validity of the simulations in this work was verified by fabricating and testing a prototype of the proposed antenna. The results of this study indicate that split ring inclusions in DRAs, especially low-permittivity DRAs, can offer significant antenna miniaturizations, without adversely affecting their performance in terms of impedance bandwidth, gain, and radiation efficiency.

## Figures and Tables

**Table 1:** Specifications of the host substrate and excitation probe.

Host Substrate Dimensions (mm)			Excitation Dimensions (mm)			Host Substrate Properties		
L	H	W	$h_{\mathrm{probe}}$	$r_{\mathrm{probe}}$	$r_{\mathrm{port}}$	$\varepsilon_{r\_\mathrm{host}}$	$\mu_{r\_\mathrm{host}}$	tan$\delta_{\mathrm{host}}$
15	7.5	0.254	7.5	0.625	2	2.2	1	0.0009

**Table 2:** SRR arrangements and corresponding SRR unit cell dimensions.

Arrangement	Geometry (Front View)	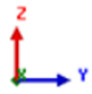	$L_{\mathrm{unit cell}}$ (mm)	$H_{\mathrm{unit cell}}$ (mm)	$L_{\mathrm{SRR}}$ (mm)	$H_{\mathrm{SRR}}$ (mm)	$N^{\;1}A^{\;2}$ (mm^2^)	${C_{\mathrm{SRR}}\;}^{3}$ (mm)
$3\;\vec{x}\times 3\;\vec{y}\times 3\;\vec{z}$	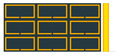		5	2.5	4.6	2.1	225.72	12.4
$3\;\vec{x}\times 3\;\vec{y}\times 2\;\vec{z}$	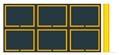		5	3.75	4.6	3.35	249.48	14.9
$3\;\vec{x}\times 3\;\vec{y}\times 1\;\vec{z}$	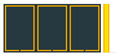		5	7.5	4.6	7.1	273.24	22.4
$3\;\vec{x}\times 2\;\vec{y}\times 2\;\vec{z}$	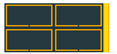		7.5	3.75	7.1	3.35	260.82	19.9
$3\;\vec{x}\times 2\;\vec{y}\times 1\;\vec{z}$	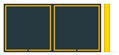		7.5	7.5	7.1	7.1	285.66	27.4
$3\;\vec{x}\times 1\;\vec{y}\times 1\;\vec{z}$	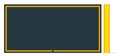		15	7.5	14.6	7.1	298.08	42.4

^1^ The number of SRRs; ^2^ the area of an individual SRR; ^3^ the circumference of an individual SRR.

**Table 3:** Antenna performance of SRR arrays with different ring arrangements.

Arrangement	$f_{r}$ (GHz)	BW (%)	η (%)	Gain (dBi)	X-Pol (dB)	Dir (dBi)	$\varepsilon_{r\_\mathrm{equivalent}}$		MC
							Cal. ^1^	Sim. ^2^	
$3\vec{y}\times 3\vec{z}$	7.1902	10.26	98.17	8.37	−18.36	8.45	91.5	90.0	6.40
$3\vec{y}\times 2\vec{z}$	5.9528	5.09	95.57	8.28	−21.14	8.48	140.2	143.7	8.08
$3\vec{y}\times 1\vec{z}$	4.1136	2.11	83.15	7.65	−26.83	8.45	308.9	335.5	12.35
$2\vec{y}\times 2\vec{z}$	4.5063	1.38	76.04	7.26	−21.84	8.45	255.1	273.8	11.16
$2\vec{y}\times 1\vec{z}$	3.3161	1.34	75.14	7.36	−28.12	8.60	482.5	536.5	15.62
$1\vec{y}\times 1\vec{z}$	2.0729	0.69	51.77	5.74	−27.25	8.60	1255.5	1435.0	25.54

^1^ Calculated $\varepsilon_{r\_\mathrm{equivalent}}$ using equations in [23]; ^2^ simulated $\varepsilon_{r\_\mathrm{equivalent}}$ using HFSS.

**Table 4:** Performance of $3\vec{x}\times 3\vec{y}\times 1\vec{z}$ antennas with different types of SRRs.

Inclusions	w (mm)	g (mm)	$f_{r}\;$ (GHz)	BW (%)	η (%)	Gain (dBi)	X-Pol (dB)	Dir (dBi)
SRR	0.2	0.2	4.1136	2.11	83.15	7.65	−26.83	8.45
CSRR	0.2	0.2	3.0925	1.16	69.25	6.60	−26.55	8.20
	0.5	0.2	3.5235	0.70	70.53	6.58	−28.22	8.10
	0.5	0.8	3.6612	0.66	69.51	6.34	−27.34	7.92
ESRR	0.2	0.2	2.4570	0.88	41.01	4.25	−26.81	8.12
	0.5	0.2	2.6924	0.65	55.61	5.63	−28.67	8.18
	0.5	0.8	3.6730	0.67	62.90	5.98	−26.63	7.99
SSRR	0.2	0.2	1.6959	0.38	17.42	0.86	−32.19	8.45
	0.5	0.2	2.0710	0.42	25.50	2.53	−32.70	8.47
	0.5	0.8	2.2059	0.49	40.50	4.44	−32.29	8.40

**Table 5:** Dimensions of the fabricated $1\;\vec{x}\times 1\;\vec{y}\times 1\;\vec{z}$ split ring antenna.

Host Substrate (mm)			SRR (mm)				Probe (mm)			Air Gap (mm)	Ground Plane (mm)	
L	H	W	$L_{\mathrm{SRR}}$	$H_{\mathrm{SRR}}$	w	g	$r_{\mathrm{probe}}$	$h_{\mathrm{probe}}$	$g_{\mathrm{probe}}$	h	$L_{g}$	$H_{\mathrm{sw}}$
15	7.5	0.127	14.6	7.1	1.6	0.2	0.625	7.5	0.35	0	124	40

## Data Availability

Data available within the article.
